# Advances in liver US, CT, and MRI: moving toward the future

**DOI:** 10.1186/s41747-021-00250-0

**Published:** 2021-12-07

**Authors:** Federica Vernuccio, Roberto Cannella, Tommaso Vincenzo Bartolotta, Massimo Galia, An Tang, Giuseppe Brancatelli

**Affiliations:** 1 Section of Radiology— Department of Biomedicine, Neurosciences and Advanced Diagnostics (BiND), University Hospital “Paolo Giaccone”, Via del Vespro 129, 90127 Palermo, Italy; 2 Department of Health Promotion, Mother and Child Care, Internal Medicine and Medical Specialties (PROMISE), University Hospital of Palermo, Via del Vespro 129, 90127 Palermo, Italy; 3 Service de radiologie, Hôpital Beaujon, APHP.Nord, Clichy, France; 4 Department of Radiology, Fondazione Istituto Giuseppe Giglio Ct.da Pietrapollastra, Via Pisciotto, 90015 Cefalù (Palermo), Italy; 5 Department of Radiology, Centre Hospitalier de l’Université de Montréal (CHUM), Montréal, Quebec Canada; 6 Centre de Recherche du Centre hospitalier de l’Université de Montréal (CRCHUM), Montréal, Quebec Canada; 7 Department of Radiology, Radiation Oncology and Nuclear Medicine, Université de Montréal, Montréal, Canada

**Keywords:** Biomarkers, Non-alcoholic fatty liver disease, Ultrasonography, Tomography, X-ray computed, Magnetic resonance imaging

## Abstract

Over the past two decades, the epidemiology of chronic liver disease has changed with an increase in the prevalence of nonalcoholic fatty liver disease in parallel to the advent of curative treatments for hepatitis C. Recent developments provided new tools for diagnosis and monitoring of liver diseases based on ultrasound (US), computed tomography (CT), and magnetic resonance imaging (MRI), as applied for assessing steatosis, fibrosis, and focal lesions. This narrative review aims to discuss the emerging approaches for qualitative and quantitative liver imaging, focusing on those expected to become adopted in clinical practice in the next 5 to 10 years. While radiomics is an emerging tool for many of these applications, dedicated techniques have been investigated for US (controlled attenuation parameter, backscatter coefficient, elastography methods such as point shear wave elastography [pSWE] and transient elastography [TE], novel Doppler techniques, and three-dimensional contrast-enhanced ultrasound [3D-CEUS]), CT (dual-energy, spectral photon counting, extracellular volume fraction, perfusion, and surface nodularity), and MRI (proton density fat fraction [PDFF], elastography [MRE], contrast enhancement index, relative enhancement, T1 mapping on the hepatobiliary phase, perfusion). Concurrently, the advent of abbreviated MRI protocols will help fulfill an increasing number of examination requests in an era of healthcare resource constraints.

## Key points

- Technical advances in liver imaging have been observed for ultrasound, computed tomography, and magnetic resonance imaging (MRI).
- Quantitative liver imaging biomarkers are promising to measure disease severity and reduce interoperator variability.
- Quantitative liver imaging biomarkers have the potential to be increasingly adopted in clinical practice.
- Abbreviated MRI protocols will help fulfill an increasing number of examination requests.

## Background

Over the past two decades, we have witnessed the high prevalence of nonalcoholic fatty liver disease (NAFLD), the advent of curative treatments for hepatitis C, the emergence of quantitative imaging, and the need for earlier detection of liver malignancies [1, 2]. These changes in the epidemiology of chronic liver disease and clinical needs have encouraged radiologists to adopt new imaging techniques.

Technical advances in liver imaging have been observed for ultrasound (US), computed tomography (CT), and magnetic resonance imaging (MRI). This narrative review aims to discuss the emerging approaches for qualitative and quantitative liver imaging based on US, CT, and MRI. While some of these techniques are not yet validated, we have focused on those that are expected to become adopted within the next 5 to 10 years in clinical practice for the assessment of diffuse liver diseases and focal liver lesions in cirrhotic, oncologic, or otherwise healthy patients. Diffuse liver diseases encompass a wide range of viral, metabolic, cholestatic, or autoimmune as well as vascular diseases. Briefly, these diffuse liver diseases may present fat, iron, inflammation, biliary, vascular, or fibrosis changes at histopathology. Significant research has been performed on imaging-based quantification of liver steatosis and fibrosis and will be discussed in this narrative review.

## Clinical needs

### Assessment of hepatic steatosis

Steatosis is characterized by an abnormal accumulation of lipids within the hepatocytes, leading to an overall hepatic-fat content greater than 5% of liver weight. At pathology, steatosis is graded from 0 to 3 based on the proportion of hepatocytes presenting macrovesicular steatosis: grade 0 (normal) < 5%; grade 1 (mild) = 5–33%; grade 2 (moderate) = 34–66%; and grade 3 (severe) ≥ 67% [3]. NAFLD is nowadays the most frequent cause of hepatic steatosis and may evolve into nonalcoholic steatohepatitis (NASH) with development of inflammation and fibrosis [4]. The reference standard for quantification of hepatic steatosis is liver biopsy. However, biopsy is invasive and vulnerable to sampling bias, especially when steatosis is distributed heterogeneously [5, 6]. The American Association for the Study of Liver Diseases guidance recommends considering liver biopsy in patients with NAFLD at increased risk of having steatohepatitis and/or advanced fibrosis [7]. Therefore, noninvasive biomarkers are needed to quantify hepatic steatosis particularly at those early stages where lifestyle changes may have a significant impact to avoid progression to fibrosis.

### Assessment of hepatic fibrosis

Hepatic fibrosis is characterized by an excessive accumulation of extracellular matrix proteins due to activation of hepatic stellate cells. This fibrotic scarring process may be observed in all causes of chronic liver diseases. The diagnosis and staging of hepatic fibrosis is crucial for the management of patients with chronic liver disease because early-stage fibrosis is potentially reversible with prompt treatment, and advanced fibrosis is an independent predictor of overall mortality [8]. Biopsy is the reference standard for staging fibrosis [9]; however, because it is invasive, several imaging-based methods have been investigated for detection and differentiation of fibrosis stages [10, 11].

### Assessment of focal liver lesions

Focal liver lesions encompass a wide range of benign and malignant lesions that require different management. The characterization of focal liver lesions must take into account the clinical background (*i.e.*, cirrhotic, oncologic, or nononcologic noncirrhotic patients) because the epidemiology and imaging presentations can substantially differ. In the setting of cirrhosis, hepatocellular carcinoma (HCC) must be excluded or diagnosed promptly [12]. In oncologic patients, high sensitivity and specificity are required for detection and diagnosis of liver metastases [13, 14]. In nononcologic noncirrhotic patients, the pretest probability of a lesion being benign is high; hence, lesion characterization should be performed at the minimum cost and with high specificity to avoid unnecessary treatment.

### From qualitative to quantitative assessment

Table 1 provides an overview of imaging techniques discussed in this narrative review. The qualitative radiological assessment has represented the main approach to liver imaging for years. Qualitative radiological assessment has been expanded with the ability to visualize microvascular flow on US and the improvement of image quality on all imaging modalities. Quantitative imaging biomarkers are also increasingly adopted in clinical practice for extraction of quantifiable features to measure disease severity and reduce inter-operator variability.

**Table 1:** Previous/current and emerging liver imaging techniques for assessing steatosis, fibrosis, and focal lesions

Imaging modality	Purpose	Previous/current techniques	Emerging techniques
Ultrasound	Steatosis assessment	B-mode	Controlled attenuation parameter (CAP) Backscatter coefficient Radiomics
	Fibrosis assessment	B-mode	Transient elastography Shear wave elastography (SWE) point SWE Radiomics
	Focal liver lesions assessment	B-mode Contrast-enhanced ultrasound (CEUS)	Novel Doppler techniques Three-dimensional CEUS
Computed tomography	Steatosis assessment	Single-energy	Dual-energy Spectral photon counting Radiomics
	Fibrosis assessment	Morphological changes	Hepatic extracellular volume fraction Perfusion Surface nodularity Radiomics
	Focal liver lesions assessment	Multiphasic contrast-enhanced	Perfusion Spectral photon counting Radiomics
Magnetic resonance imaging	Steatosis assessment	Spectroscopy	Proton density fat fraction (PDFF) Radiomics
	Fibrosis assessment	Morphological changes	Elastography Contrast enhancement index Relative liver enhancement T1 mapping on hepatobiliary phase Perfusion
	Focal liver lesions assessment	Multiphasic contrast-enhanced	Abbreviated protocols Radiomics

## Ultrasonography

Brightness-mode (B-mode) US remains the mainstay for anatomical imaging but several new techniques have been introduced, such as elastography and quantitative US parameters for assessment of liver tissue properties other than echogenicity and new Doppler and contrast-enhanced (CEUS) modes for vascular assessment.

### Assessment of hepatic steatosis

**Fig. 1:**
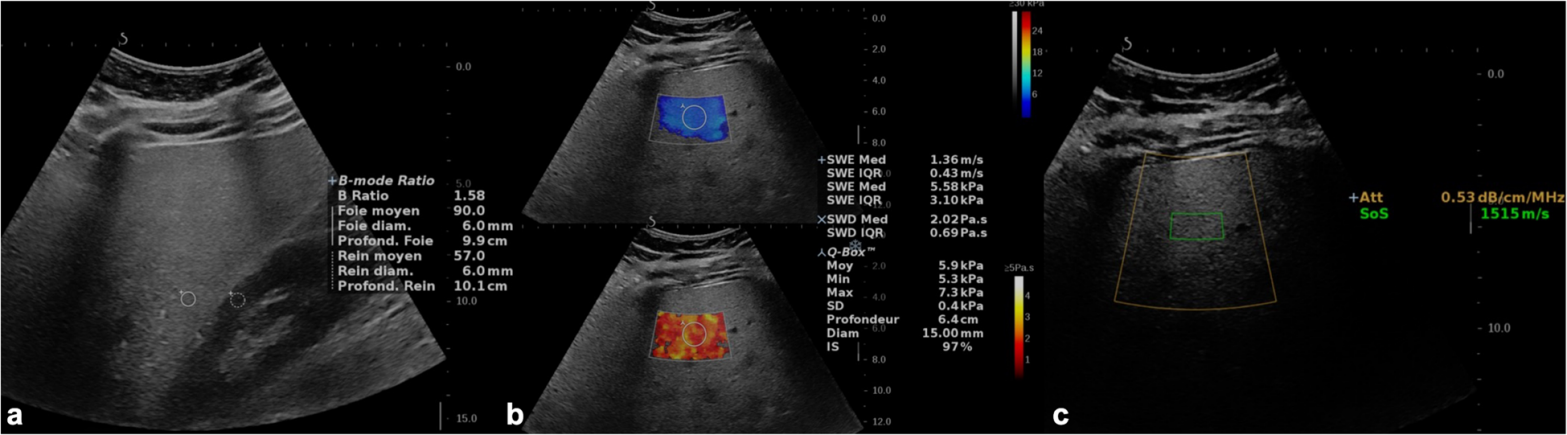
40-year-old man with hepatic steatosis. Multiparametric ultrasound assessment includes (**a**) hepatorenal index, (**b**) shear wave elastography imaging, and (**c**) attenuation imaging coefficient and sound speed estimation

Quantitative US techniques characterize tissue microstructure by measuring acoustic parameters. In the last decades, controlled attenuation parameter (CAP), attenuation imaging coefficient, and sound speed estimation on US showed to be promising for monitoring steatosis severity in chronic liver diseases with moderate to high sensitivity and specificity for diagnosing different grades of steatosis (Fig. 1) [15–17]. CAP is among the most widely studied quantitative technique for assessment of liver steatosis. CAP measures the attenuation of ultrasound echoes through the liver and expresses the acoustic energy attenuation in decibel/meter (dB/m). CAP software is available on the transient elastography device (FibroScan, Echosens, France) and measured simultaneously to liver stiffness [18]. CAP is, however, limited by the lack of a B-mode guidance to identify the sampling area. To overcome this limitation, software for measuring attenuation imaging coefficient with B-mode guidance has been proposed by several US vendors [19].

The use of radiomics for staging hepatic steatosis has been recently investigated. Tang et al. [20] developed a machine learning model based on quantitative US parameters combined with elastography in a preclinical study in rats, and demonstrated moderately high accuracy of the model in the differentiation of the different grades of hepatic steatosis and inflammation grades.

### Assessment of hepatic fibrosis

**Fig. 2:**
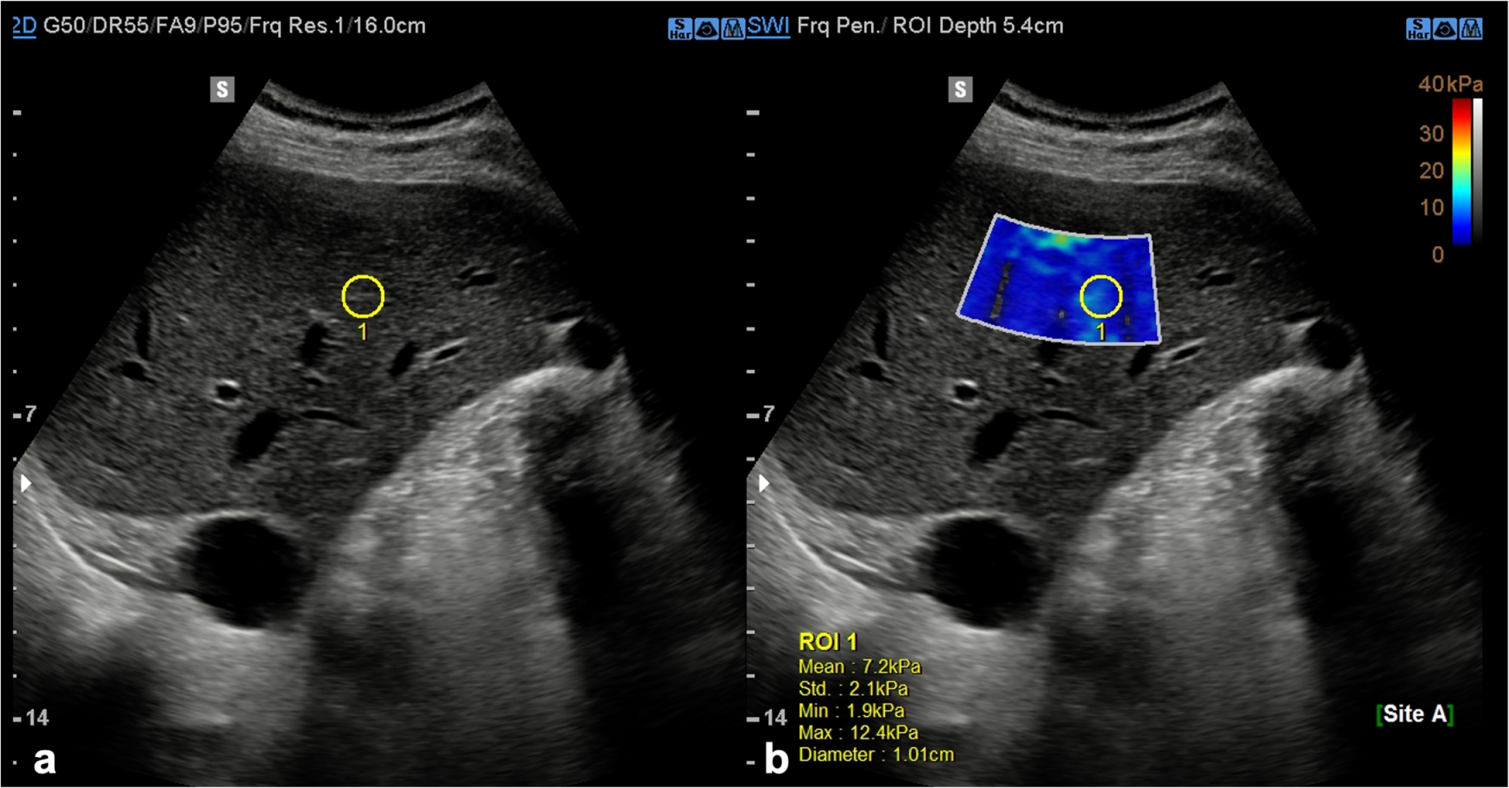
Ultrasound shear wave elastography for assessment of hepatic fibrosis. **a** Brightness-mode image with a 1.0-cm circular region of interest indicating a mean stiffness of 7.2 kPa. **b** Shear wave imaging mode indicating the liver stiffness with a color parametric map along a scale from 0 to 42 kPa

US elastography provides a widely available, noninvasive, low cost, and repeatable method to assess liver fibrosis, and it is recommended in diagnostic work-up of chronic liver diseases [21]. It enables quantitative assessment of liver stiffness by applying an external force by means either of a mechanically induced impulse (as in transient elastography, TE) or US-induced focused radiation force impulse (as in point shear wave elastography, pSWE) and measuring the velocity of propagated US waves axial (in TE) or perpendicular (in pSWE quantification) to the US beam pathway [21]. Depending on the implementation, various parameters related to tissue stiffness such as shear wave speed or young elastic modulus are reported as biomarkers of liver fibrosis (Fig. 2) [22]. Recent developments of US elastography include volumetric assessment of liver stiffness and its variation in real time. One limitation impeding the clinical use of US elastography is that cutoff values for fibrosis staging vary across US systems from different vendors. In general, however, a Young modulus of less than 7 kPa (1.5 m/s) (pSWE and 2D SWE) can help rule out significant fibrosis [23].

The use of radiomics for staging hepatic fibrosis on US, CT, or MRI has been investigated in the last decade, with most studies including patients with hepatitis C or B infection as etiology of the chronic liver disease and only few patients with NAFLD [24–26]. In regard to radiomics applied to 2D-SWE, a prospective study including 398 patients with chronic hepatitis B identified a predictive radiomics model with excellent area under the ROC curve (AUC) for both cirrhosis and advanced fibrosis [24].

### Assessment of focal liver lesions

**Fig. 3:**
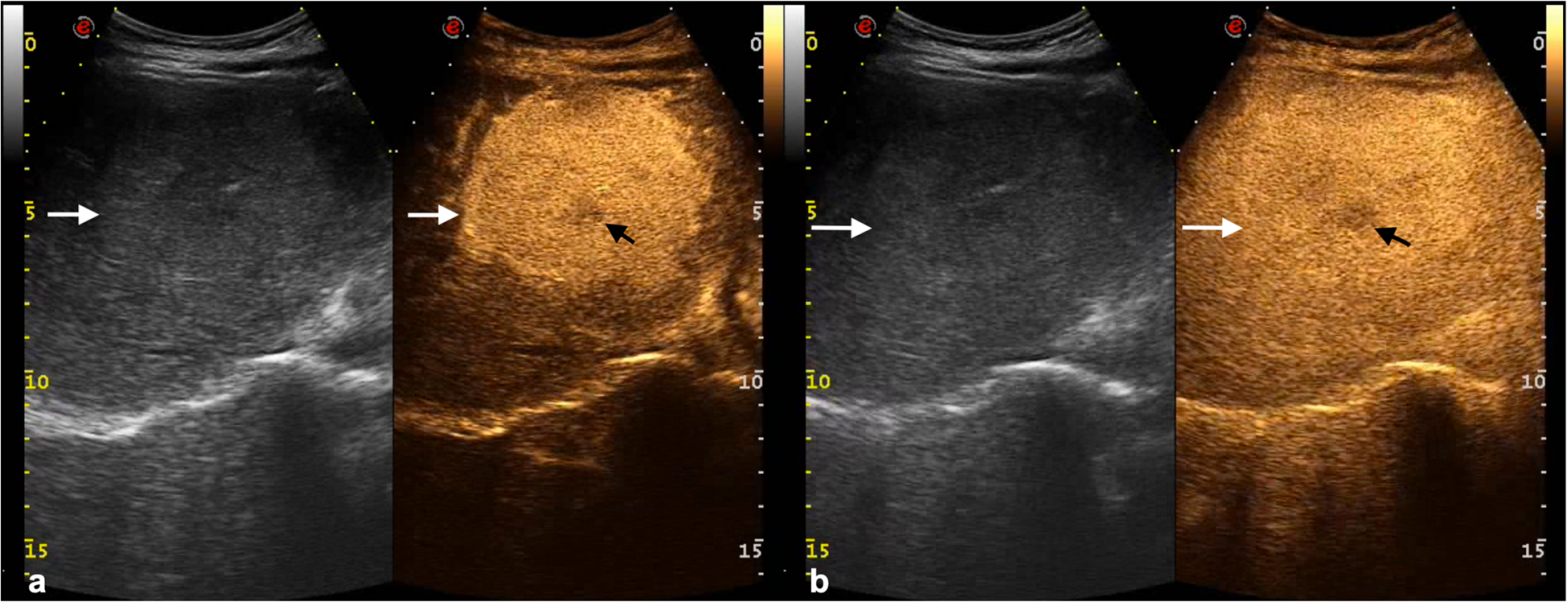
Contrast-enhanced ultrasound for assessment of focal nodular hyperplasia. Dual-display images with brightness-mode (left) and contrast-enhanced ultrasound images (right). **a** Images in arterial phase (28 s after the i.v. injection of contrast agent) show homogeneous and strong enhancement (white arrows) with a central hypoechoic scar (black arrow). **b** In the portal venous phase (86 s after the injection), the lesion is still slightly hyperechoic to the adjacent liver parenchyma (white arrows) and the central scar remains hypoechoic (black arrow)

CEUS is the most economically appropriate second-line imaging modality for the characterization of focal liver lesions after inconclusive baseline US in nononcologic noncirrhotic patients [27, 28]. The main feature indicating benignity is a sustained and prolonged contrast enhancement with lack of washout in the portal venous and late phases (Fig. 3) [27]. The main caveat to this observation is that well-differentiated HCC may show prolonged and sustained contrast-enhancement too, although the clinical setting is different. In cirrhotic patients, CEUS allows characterization of contrast enhancement patterns of HCC with good sensitivity and specificity, without the use of ionizing radiation and with a much higher temporal resolution than CT or MRI [29]. CEUS can also be used to guide locoablative therapies and to assess treatment response [30]. In oncologic patients, CEUS provides higher sensitivity compared to unenhanced US for the detection of liver metastases and for characterization of liver lesions deemed indeterminate on CT and MRI [31]. In patients treated with antiangiogenic therapies for solid tumors, data show encouraging results in the use of dynamic CEUS to distinguish responders from non-responders [32]. In addition, microbubbles are being actively studied not only as US contrast agents but also for local drug delivery under US triggering in animal studies [33].

**Fig. 4:**
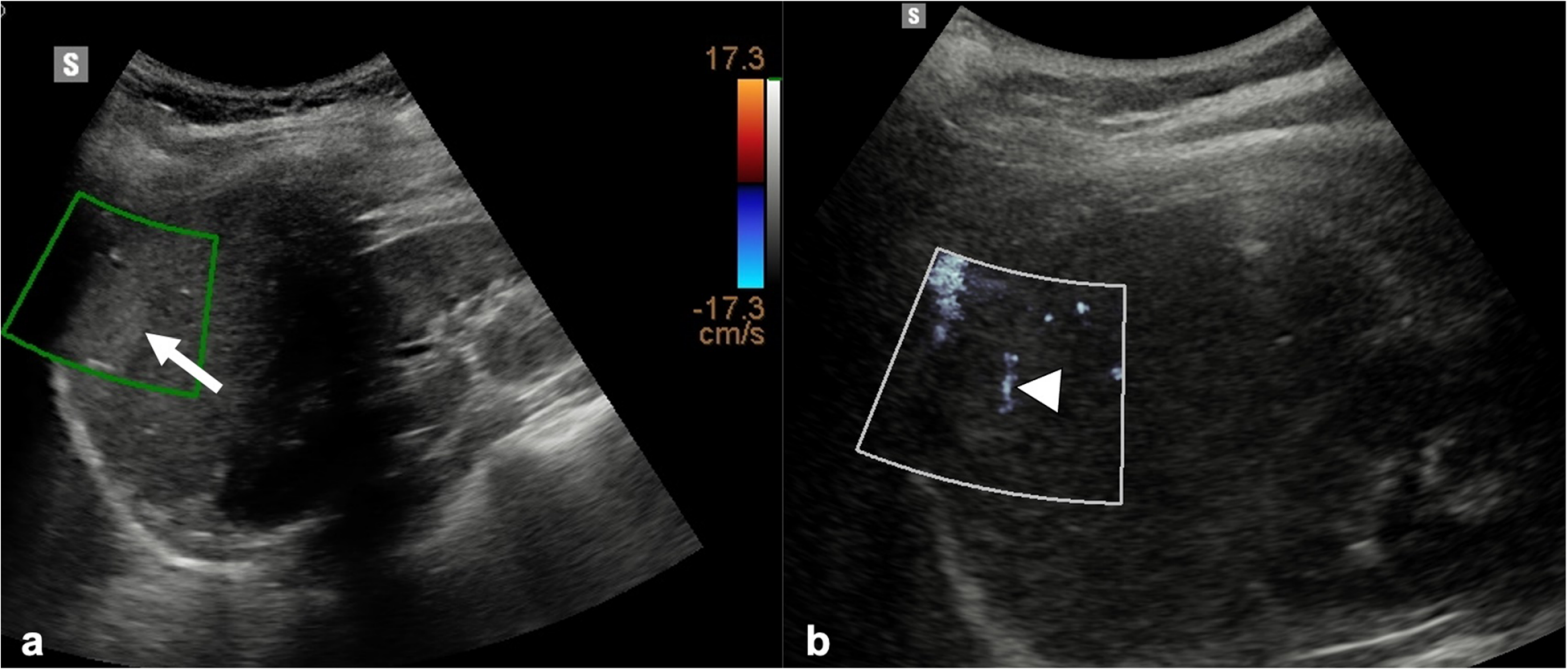
Microvascular flow imaging in a young woman with focal nodular hyperplasia. **a** Brightness-mode ultrasound demonstrates a 2.8-cm focal nodular hyperplasia (arrow) lacking any vascularization at conventional color Doppler. **b** Microvascular assessment with microvascular flow imaging clearly depicts an intralesional vessel (arrowhead)

Novel Doppler techniques assess microvascular flow by differentiating the signal of slow or small vessels from clutter artifacts. US manufacturers have introduced techniques such as superb microvascular imaging (SMI, Canon Medical Systems, Otawara, Japan), microflow imaging (MFI, Philips Healthcare, Best, The Netherlands), and microvascular flow imaging (MVFI) (MV-FlowTM, Samsung Medison Co., Ltd., Seoul, Korea). These new, third-generation Doppler-based techniques enable the depiction of slow blood flow at a high spatial resolution and frame rate by using advanced clutter suppression, thus improving sensitivity and accuracy of Doppler US in the detection of vascularity in liver tumors with a safe, inexpensive, and readily available modality (Fig. 4) [34, 35]. Dynamic three-dimensional CEUS allows the evaluation of tumor perfusion in three orthogonal planes and the detection of flow in vessels as tiny as 40 μm with quantification of tumor contrast enhancement from time-intensity curves.

Radiomics of US images may be also a promising technique for assessment of focal liver lesions. Liu D et al. [36] recently proposed a radiomic-based CEUS learning model to predict response of HCC patients to their first transarterial chemoembolization session by quantitatively analyzing their preoperative CEUS examinations. However, prospective multicenter studies on large study populations with validation cohorts are still needed before considering the clinical adoption.

## Computed tomography

CT represents a mainstay for liver assessment, with the vast majority of acquisitions performed with single-energy CT scans. The great benefit in identifying a reliable method to quantify hepatic steatosis or fibrosis on CT would be the far wider adoption of CT compared to MRI for many clinical purposes. Contrast-enhanced CTs in portal venous phase are commonly performed in oncologic patients while multiphase CTs with late arterial, portal venous, and delayed phases are performed for characterization of focal liver lesions. Quantification on single-energy CT images has been based for years on size and density measurements [37]. Emerging techniques include dual-energy CT (DECT), post-processing software, perfusion CT (pCT), and photon-counting detector CT (PCD-CT).

### Assessment of hepatic steatosis

**Fig. 5:**
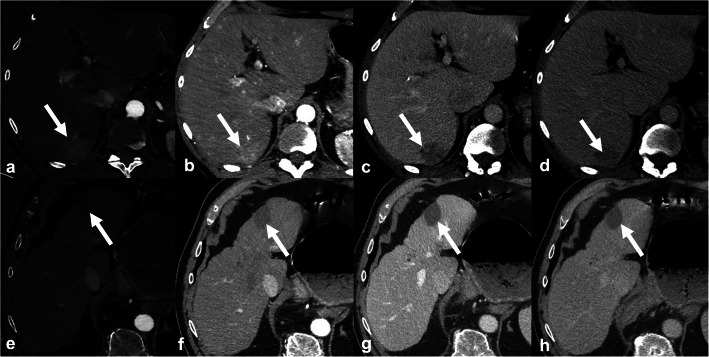
Dual-energy computed tomography (DECT). **a**-**d** 58-year-old man with 39-mm hepatocellular carcinoma (arrows) imaged with DECT. Arterial phase hyperenhancement is better visualized on iodine map (**a**) than on the standard late hepatic arterial phase (**b**). Lesion also shows washout on portal venous (**c**) and delayed phase (**d**). **e**-**h** 79-year-old man with hepatocellular carcinoma treated with microwave ablation (arrows) and imaged with DECT. Iodine map (**e**), standard late hepatic arterial phase (**f**), portal venous (**g**), and delayed phase (**h**) demonstrate no residual enhancement consistent with complete tumor treatment

DECT overcomes a key limitation of single-energy CT which relies on Hounsfield units for quantification. DECT is based on CT data acquisition at two different energy spectra [38]. Post-processing of DECT data yields several types of images including monochromatic image reconstructions that are particularly useful to improve iodine contrast visualization, attenuation maps of different elements such as iodine (Fig. 5), calcium, and water on the basis of their atomic number, and virtual unenhanced series—obtained by virtually removing the iodine from enhanced images—which may obviate the need for acquisition of an unenhanced series [38]. The spectral curve for hepatic steatosis increases in the attenuation of fat with an increase in tube potential due to the decreased attenuation at lower energy levels in presence of fat content [38]. However, material decomposition with DECT does not seem to improve the accuracy of fat quantification over single energy CT on unenhanced CT, while the adoption of the multimaterial decomposition algorithm on contrast-enhanced DECT might be promising for quantification of fat volume fraction from the contrast-enhanced scan eliminating the need for a separate unenhanced CT scan [38, 39].

Few radiomics studies have been performed for assessment of hepatic steatosis on CT so far. In patients without suspicion of fibrosis, unenhanced CT texture analysis may predict NASH with an AUC of 94%, but this AUC drops to 60% in patients with suspected fibrosis [40]. Deep learning-based automated segmentation tools at unenhanced and contrast-enhanced CT have been used for quantifying liver fat at a population level demonstrating objective correlation with manual measurement of fat attenuation, but with lack of pathology or MRI PDFF as reference standard [41].

### Assessment of hepatic fibrosis

Quantification of hepatic extracellular volume fractions on the equilibrium phase in routine liver contrast-enhanced CT is an emerging technique for assessment of liver fibrosis, but still needs extensive validation [42].

Liver pCT is theoretically a good technique to stage fibrosis because it captures perfusion changes occurring during fibrosis [43]. Liver pCT is performed through acquisition of serial images at high temporal resolution after intravenous administration of a bolus of iodinated contrast agent [43]. Images are then post-processed to compute quantitative or semiquantitative tissue perfusion parameters, such as blood flow, blood volume, mean transit time, portal liver perfusion, arterial liver perfusion, and hepatic perfusion index [43]. The main limitations of liver pCT are related to radiation exposure, for which different technical solutions and protocols aimed at reducing radiation dose are still under investigation [44], to the wide variability of image acquisition protocols and analysis methods which limit the comparison of perfusion parameters between vendors and to the need for significant post-processing. All these limitations have limited the adoption of liver pCT in clinical practice.

**Fig. 6:**
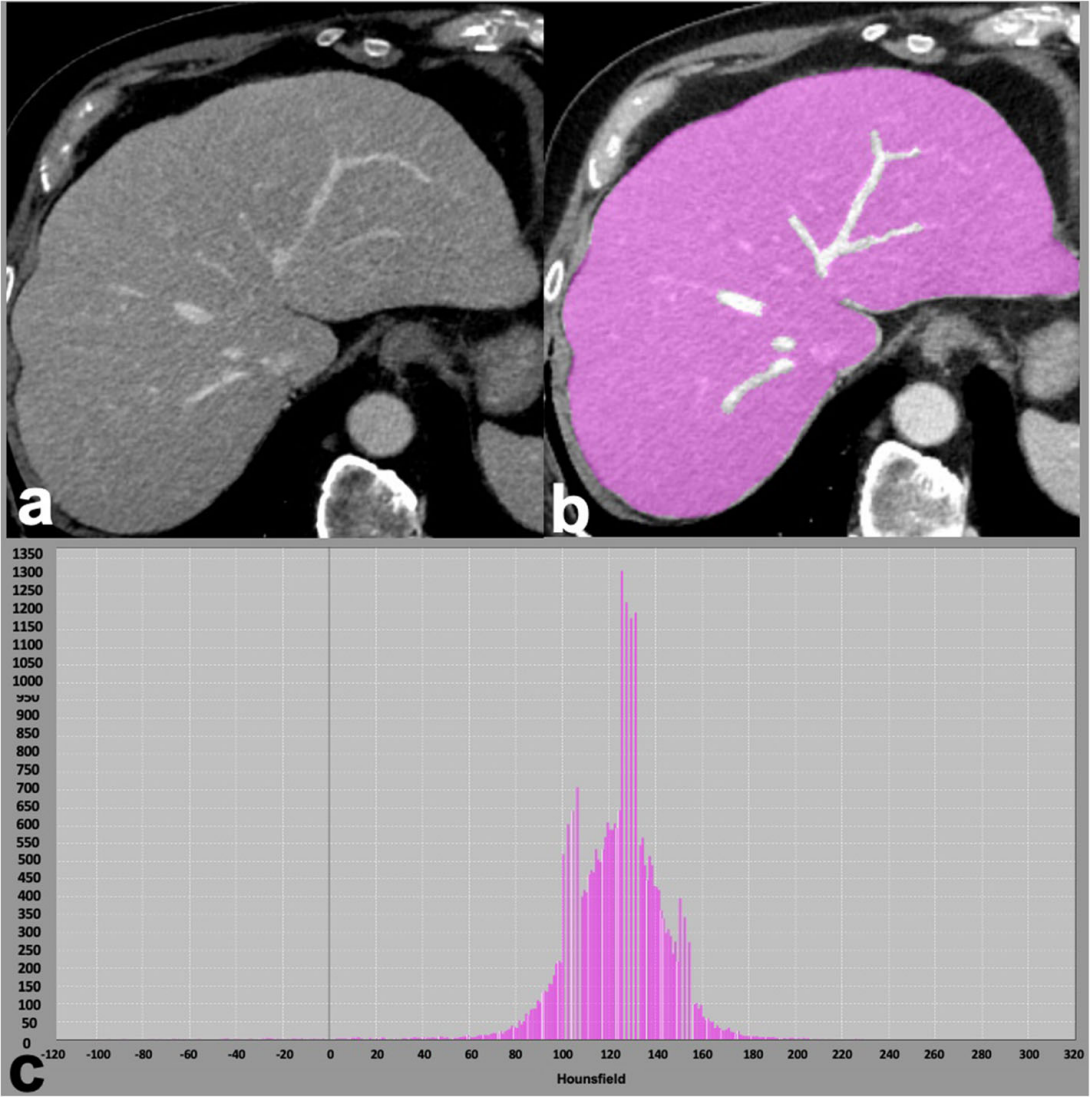
79-year-old man with nonalcoholic fatty liver disease and histopathologically proven advanced fibrosis (stage F3). Contrast-enhanced CT in portal venous phase (**a**) shows a dysmorphic liver with mild lobulations. Whole liver segmentation was performed (**b**), excluding major hepatic vessels, to extract radiomics features using a free software (LIFEx, www.lifexsoft.org). The corresponding histogram (**c**) shows the distribution of pixel intensities within the segmented region of interest

Postprocessing software has been developed to extract quantitative data from CT scans for liver imaging to quantify the hepatic morphologic fibrotic changes occurring in chronic liver diseases. Several promising CT techniques have been proposed for staging hepatic fibrosis with post-processing software, including quantitative measures of liver surface nodularity and the liver segmental volume ratio as well as the combination of laboratory values, liver surface nodularity, and radiomics (Fig. 6) [45–48]. These techniques will require further investigation to ensure repeatability across different CT acquisition and reconstruction parameters, different CT scanners, and in patient populations prior to clinical validation [46, 48, 49]. Among all these techniques, liver surface nodularity is most likely to be adopted soon given the promising results reported, rapid image processing, absence of requirement for patient fasting or for additional hardware, very low technical failure rate, and vendor-neutral results which allow applicability with all CT scanners already available. We anticipate that some of these tools will be integrated into CT workstations or PACS systems to provide multiparametric quantitative assessment of liver parenchyma [50].

In regard to radiomics, Choi et al. [48] developed a deep learning system on portal venous phase CT images in 7461 patients achieving an AUC of 0.96, 0.97, and 0.95 for ≥ F2, ≥ F3, and F4 respectively. The results of radiomics studies are, therefore, very promising for noninvasive staging of hepatic fibrosis but still not generalizable due to dependencies on scanner, vendor, acquisition, and reconstruction protocol. Hence, external validation in larger cohorts with different causes of chronic liver disease will be required [47, 49].

### Assessment of focal liver lesions

Contrast-enhanced CT may permit definite characterization of benign liver lesions that are indeterminate at US in nononcologic noncirrhotic patients. In cirrhotic patients, contrast-enhanced CT and MRI are the current recommended techniques for diagnosis and post-treatment follow-up of HCC [12]. However, CT is commonly preferred to MRI in clinical practice due to access and time constraints, despite its lower sensitivity [51]. In oncologic patients, CT is widely used for staging and follow-up of many malignancies, with the main diagnostic limitations represented by detection and characterization of small hypoattenuating liver lesion and detection of lesions in hepatic steatosis, which may be primary or secondary to chemotherapy [52, 53]. DECT improves diagnostic accuracy for both detection and characterization of small hypoattenuating indeterminate liver lesions as compared to single-energy CT through iodine quantification [54].

The adoption of liver pCT has been investigated in oncologic and cirrhotic patients for early detection of tumors, assessing disease prognosis based on tumor vascularity, monitoring therapeutic effects of various treatment regimens including antiangiogenic drugs, and early identification of tumor recurrence after treatment [55]. This is because liver pCT parameters correlate well with the presence and extent of tumor vessels [55]. However, as stated above, the presence of many limitations of liver pCT has limited its adoption in clinical practice.

Spectral PCD-CT has recently become technologically feasible for true multi-energy CT scanning, including for liver imaging [56]. The adoption of photon-counting detectors enables the analysis of each photon by dividing them into multiple energy bins, thus simultaneously sampling the energy spectrum at multiple regions. This allows obtaining K-edge imaging to generate maps to differentiate several elements or contrast agents at once [56]. As an example, spectral PCD-CT could allow for a dual-contrast single-scan liver protocol thus potentially improving focal liver lesion detection and characterization, with a reduced radiation exposure [56]. Despite its theoretical advantages, PCD-CT is still in its infancy, limited to preclinical or small *in vivo* studies in volunteers. Adoption in clinical practice for liver imaging will require clinical studies and validation over a longer time horizon.

**Fig. 7:**
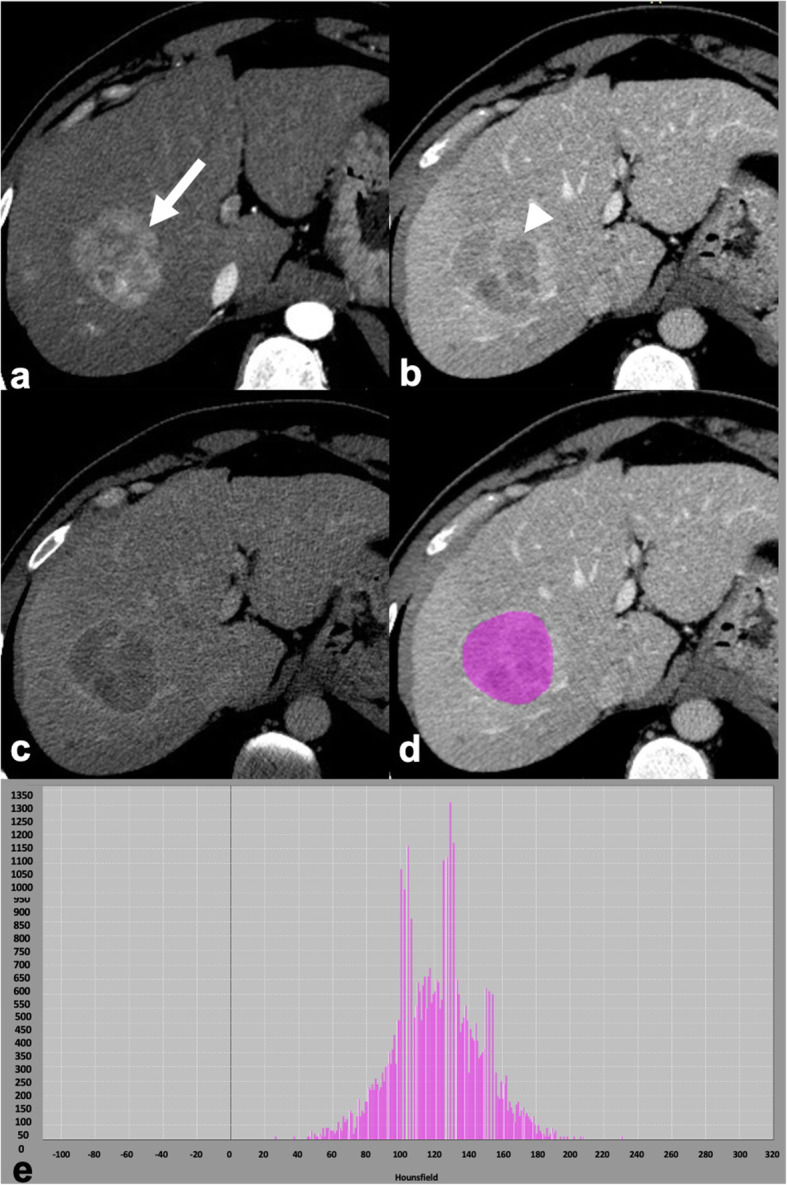
Computed tomography (CT) texture analysis in a 34-year-old man with chronic hepatitis B and 50-mm hepatocellular carcinoma. Contrast-enhanced CT shows hyperenhancement in the late arterial phase (**a**, white arrow), washout in portal venous phase (**b**, white arrowhead), and in delayed phase (**c**); a capsule is visible in delayed phase (**c**). The tumor was segmented on the portal venous phase by manually drawing a region of interest within the lesions margin (**d**), using a free software (LIFEx, www.lifexsoft.org), the corresponding histogram shows distribution of signal intensities within the region of interest (**e**)

Quantitative imaging features extracted through CT texture analysis (Fig. 7) could provide a more robust classification of indeterminate focal liver lesions or help in the response prediction after locoregional treatments. In the setting of cirrhosis and oncologic patients, deep learning radiomics CT-based models proved useful for different outcomes such as increasing the accuracy for lesion detection (HCC in cirrhosis and occult metastases in oncologic patients) as well as to predict response after treatments and overall survival [57–59]. Of note, most radiomics studies on oncologic patients focused on the use of radiomics for colorectal liver metastases on contrast-enhanced CT. In the nononcologic and noncirrhotic patient, one of the main challenges is represented by the differential diagnosis between hepatocellular adenomas and focal nodular hyperplasia and texture analysis seems to improve it [60]. Although the clinical application of radiomics and artificial intelligence for focal liver lesions is expected in the future, there are still many limiting factors, including the dependency of the radiomics analysis from vendor, equipment, imaging acquisition, or reconstruction algorithms, as well as the need for prospective validations of artificial intelligence algorithms in different study populations, which highlights the need for a cooperative worldwide effort to join clinical and imaging data [47, 49, 61].

## Magnetic resonance imaging

**Fig. 8:**
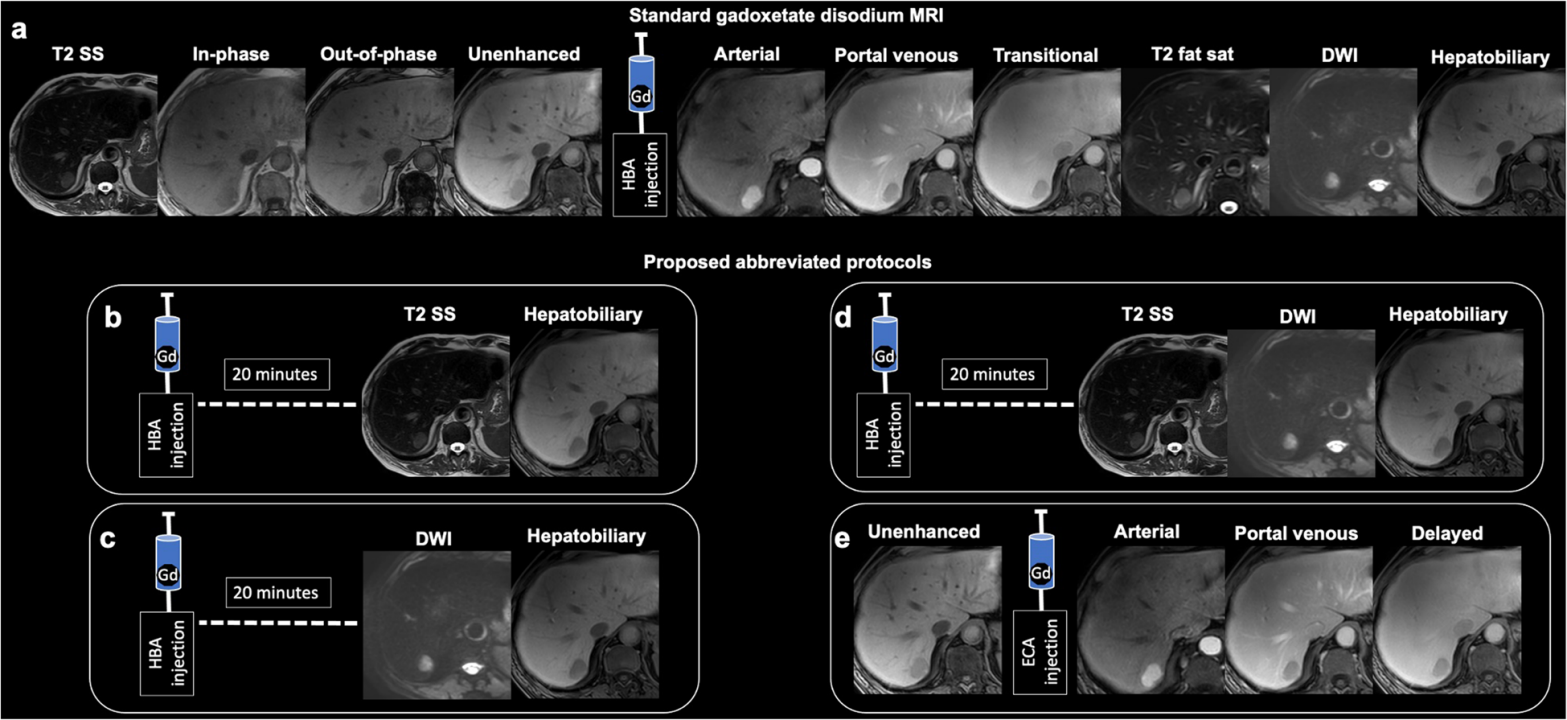
Magnetic resonance imaging of the liver. Standard and proposed abbreviated protocols. 66-year-old man with 30-mm hepatocellular carcinoma (HCC) imaged with standard gadoxetate disodium protocol (**a**) and proposed abbreviated protocols for HCC screening. Gadoxetate disodium may be administered outside the scanner 20 min before abbreviated protocols that contain a hepatobiliary phase (**b-d**) or an extracellular agent can be administered before dynamic phases (**e**). Abbreviated protocols may include T2-weighted single-shot (SS) and hepatobiliary phase (HBP) (**b**); T2-weighted SS, diffusion-weighted imaging (DWI), and HBP (**c**); DWI and HBP (**d**); or pre-contrast and arterial, portal venous, and delayed phases (**e**). ECA, extracellular contrast agent; HBA, hepatobiliary contrast agent

An adequate MRI liver protocol has to be short, comprehensive, standardized, and must guarantee reproducibility and consistency of image quality and diagnostic information (Fig. 8) [53, 54]. Extracellular and hepatobiliary contrast agents may both be used for multiphase imaging. However, hepatobiliary contrast agents provide the additional ability to acquire images in a hepatobiliary phase [62–64].

### Assessment of hepatic steatosis

MRI is considered the gold standard imaging technique for hepatic fat quantification. MRI techniques estimate the signal fat fraction by exploiting the differences in resonance frequency of water and fat proton signals. Hepatic fat quantification is achievable with MRI-based techniques that measure the proton density fat fraction (PDFF) which represents the fraction of mobile protons in liver attributable to fat. To measure the unconfounded PDFF, MRI techniques must account for T1 bias, T2 relaxation, T2* bias, and spectral complexity of fat [65]. MR spectroscopy (MRS) was previously considered the noninvasive reference standard for liver fat quantification [66]. However, MRS is time consuming and limited to one voxel which can lead to misinterpretation in cases of heterogeneous steatosis. To address these limitations, multi-echo gradient-recalled echo sequences that may cover the entire liver within one breath-hold have emerged as an accurate MRI alternative for PDFF quantification [67].

**Fig. 9:**
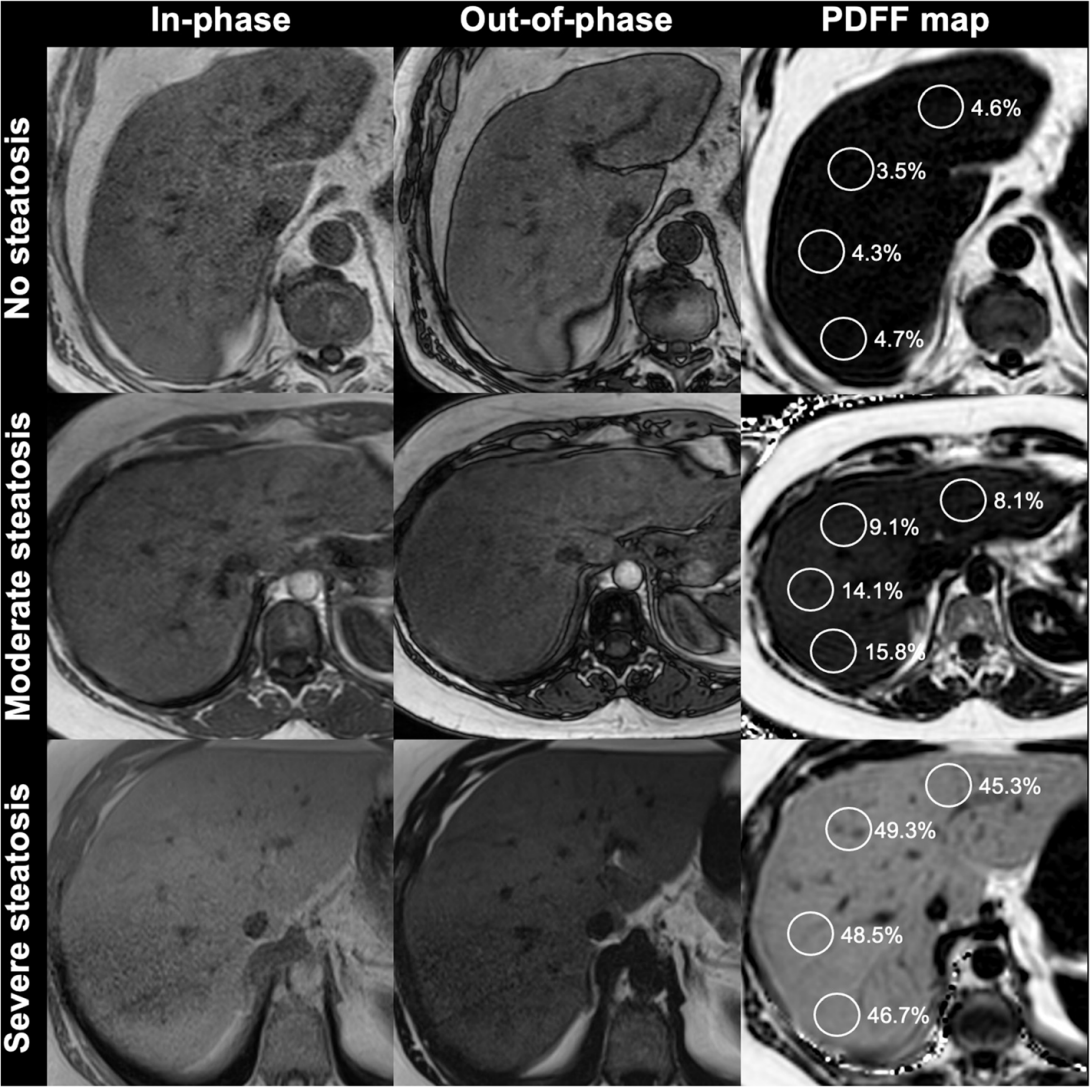
Liver fat detection and quantification in patients with nonalcoholic steatohepatitis undergoing in- and out-of-phase magnetic resonance imaging sequences (first and second columns, respectively), and proton density fat fraction (PDFF, third column). Top row: 78-year-old man without hepatic steatosis. Note the lack of signal drop on out-of-phase image and PDFF of only 3–4%. Middle row: 39-year-old woman with moderate hepatic steatosis. Note the minimal signal drop on out-of-phase image and PDFF from 8 to 16%. Bottom row: 48-year-old man with severe hepatic steatosis. Note the marked signal drop on out-of-phase image and PDFF from 45 to 49%

Nowadays, MRI-PDFF with multi-echo chemical-shift-encoded sequence is routinely used at different centers to quantify hepatic steatosis with excellent diagnostic value for classification of histologic steatosis in patients with NAFLD (Fig. 9) [67, 68]. The clinical usefulness of MRI PDFF has been demonstrated also in the context of bariatric surgery because radiology may help to identify those patients that will more likely benefit from bariatric surgery. Indeed, as demonstrated by Pooler et al. [69], bariatric patients with marked hepatic steatosis at baseline as quantified by MRI PDFF may have substantial improvement in liver PDFF regardless of starting anthropometrics or degree of weight loss following surgery. In addition, MRI-PDFF seems an adequate fat quantification biomarker in living liver donor candidates with sufficient negative predictive value for excluding clinically significant hepatic steatosis obviating the need for liver biopsy [70].

To date, not many studies assessed the role of radiomics applied to MRI for assessment of hepatic steatosis. From a prospective study by Gutmann et al. [71], it is evident that MRI radiomics applied to T1-weighted dual-echo Dixon relative fat water content may help in the prediction of type 2 diabetes mellitus and metabolic syndrome. Further research studies are expected to provide very good results in the next few years related to the use of radiomics and artificial intelligence for hepatic fat quantification, and once validated in multiple different large cohorts and across different vendors and acquisition protocols, it is expected that these techniques will be integrated in clinical routine in the long term.

### Assessment of hepatic fibrosis

MRI-based techniques for assessment of liver fibrosis include magnetic resonance elastography (MRE), diffusion-weighted imaging (DWI), MRI with gadoxetate disodium (Primovist in Europe, Eovist in the USA, Bayer HealthCare), MRI perfusion, and quantitative T1, T2, T1 rho mapping, which are still being investigated in research environments.

The leading MRI technique for staging hepatic fibrosis is MRE [72]. MRE measures liver stiffness that is directly related to the stage of fibrosis [73] allowing to differentiate the various stages with moderately high accuracy (84–92%) [74]. This technique, requires a driver to generate mechanical waves, a phase-contrast pulse sequence with motion-encoding gradients and post-processing software to obtain wave images, and inversion algorithms to generate quantitative maps of liver stiffness measurements known as elastograms [73]. MRE is commercially available on clinical scanners. Failure of MRE is known to potentially occur in patients with hepatic iron deposition secondary to the low parenchymal signal, or when using 1.5-T MRI scanners, in patients with massive ascites or high body mass index or in uncooperative patients who cannot hold their breath [73, 74].

In regard to DWI, the two most common approaches for the assessment of liver fibrosis include the quantification through ADC values extracted using a monoexponential model or an intravoxel incoherent motion analysis [75]. However, conflicting results for accuracy of DWI in staging liver fibrosis have been obtained so far, with this sequence having many technical limitations (*e.g.*, ADC values dependency on MRI scanner field strength, DWI signal being affected by hepatic iron deposition, and image quality being lowered in uncooperative patients or in patients with ascites).

The assessment of hepatocellular function in the hepatobiliary phase on gadoxetate disodium-enhanced MRI has been investigated as a surrogate biomarker for estimation of liver fibrosis and outcomes in patients with chronic liver disease [76, 77]. This may be obtained by using different quantitative parameters such as contrast enhancement index, relative liver enhancement, and T1 mapping of hepatobiliary phase images [76, 77]. Indeed, progressive fibrotic changes in hepatic uptake of the hepatobiliary contrast may result from either decreased expression of hepatic organic anion transporters due to hepatocyte dysfunction or degeneration or prolongation of liver enhancement due to decreased biliary excretion.

Perfusion MRI refers to imaging of tissue blood flow typically performed using T1-weighted MRI sequences after contrast agent injection employing a high temporal resolution technique that allows for repeated imaging of the same area in the liver about every 4 s [43]. Perfusion MRI might be useful for staging fibrosis with preliminary good results, but this technique is still limited by breath motion artifacts, variability dependent on acquisition protocols, reconstruction methods, and of course the software used for quantitative analysis, so that there is still not a unique validated acquisition protocol [43]. T1, T2, and T1 rho mapping technology is being also investigated because with progressive hepatic fibrosis, excessive accumulation of extracellular matrix proteins occurs and oftentimes is associated with coexistent inflammation and high water content, leading to prolongation of T1 and T2 relaxation times in fibrotic tissues [78].

Finally, in regard of radiomics, a predictive MRI model of Park et al. [25] obtained on hepatobiliary phase images from 329 patients demonstrated an AUC of 0.89–0.91 for staging advanced hepatic fibrosis.

### Assessment of focal liver lesions

MRI is particularly helpful for the differential diagnosis between focal nodular hyperplasia and hepatocellular adenoma in nononcologic noncirrhotic patients, and has a higher sensitivity than CT in cirrhotic and oncologic patients [51, 79]. To overcome the issue of long acquisition times required by MRI, abbreviated MRI strategies have been investigated for cirrhotic and oncologic patients.

Three abbreviated MRI strategies have been investigated for HCC screening in cirrhotic patients: (1) unenhanced, (2) dynamic contrast-enhanced, and (3) hepatobiliary phase contrast-enhanced MRI. Different types of abbreviated protocols have been suggested, including axial contrast-enhanced T1 weighted with fat saturation in the hepatobiliary phase at 20 min after gadoxetate disodium and either axial T2-weighted single shot sequence or DWI and axial T1 weighted with fat saturation in the unenhanced, arterial, portal venous, and delayed phases (Fig. 4) [80, 81]. Although it seems that the diagnostic accuracy for HCC remains acceptable for clinical practice when using abbreviated liver MRI protocols [82], the differentiation between HCC and non-HCC malignancies and the identification of tumor in vein may be challenging with abbreviated protocols. Additional imaging features are being investigated to improve the differential diagnosis between HCC and non-HCC malignancies [83, 84].

MRI in oncologic patients is often a problem-solving tool in case of focal liver lesions deemed indeterminate on CT, allowing to characterize almost 60% of the cases [85, 86]. Abbreviated liver MRI protocol including DWI and hepatobiliary phase have been investigated for the routine follow-up of some oncologic patients, such as those with colorectal cancer [80, 86]. We anticipate that abbreviated liver MRI protocols will be increasingly used in an oncologic setting to shorten the MRI examination time and to leverage the higher detection sensitivity of MRI compared to other imaging modalities. It is our belief that the objectives of detecting liver metastases early during preoperative planning and monitoring the size of liver metastases in response to chemotherapy can both be achieved with abbreviated MRI protocols.

Similarly to CT, quantitative imaging features extracted through MRI texture analysis could provide a more robust classification of indeterminate focal liver lesions or help in the response prediction after locoregional treatments and some studies have been published for this purpose so far [47, 87–89]. However, most of the literature is limited to single center retrospective cohorts with insufficient number of included patients and lesions, and, therefore, the use of MRI based radiomics remains investigational.

## Clinical application of liver imaging biomarkers: open issues

Clinical application of imaging biomarkers in routine practice is still at its beginning. Most research studies on radiomics and other advanced liver imaging techniques are limited by lack of standardization and validation, thus, considered as proof-of-concept, preclinical, or exploratory studies [90, 91]. Several factors may slow the clinical adoption of imaging biomarkers including concerns about reproducibility, such as segmentation reproducibility (to be assessed with multireader segmentation), imaging data reproducibility (to be assessed with phantom studies and test-retest analysis), computational and statistical reproducibility (evaluating overfitting, controlling outliers), and research reproducibility (not easy to assess due to limited access to open data sets and to the model equation or code) [90, 92, 93]. Quantitative imaging techniques and radiomics are affected by acquisition and reconstruction settings, to the point of being not reproducible. Therefore, there is the need of identification of stable, standardized radiomic features that can be used with different scanners and imaging protocols [49].

The universal adoption of the Hounsfield scale on every CT scanner worldwide (except cone beam CT) provides a template for successful standardization, thanks to a clear definition and availability of calibration phantoms. A similar standardization should be achieved with most recent quantitative imaging techniques for the quantification of steatosis and fibrosis.

Reproducibility must be investigated in different settings and populations [94]. As in the case of other biometric data, what is considered normal for one population, might not be applicable in another population. Therefore, external validation in a different population is a prerequisite, but it is still lacking in many studies. One of the ways to overcome this issue may be to require the inclusion of the dataset along with the manuscript as a prerequisite for publication. Ensuring reproducibility, standardization, and external validation of emerging quantitative imaging biomarkers will facilitate their clinical adoption.

## Conclusions

Several advances in liver imaging have improved acquisition techniques, introduced new tissue contrast mechanisms, and provided an emerging role for quantitative biomarkers in US, CT, and MRI. The advent of abbreviated MRI protocols will help fulfill an increasing number of examination requests in an era of healthcare resource constraints. New imaging biomarkers such as PDFF are already adopted in clinical practice, and others are almost ready to be implemented with the possibility to be available in the routine clinical assessment in the near future of liver imaging. We anticipate that radiomics and artificial intelligence will enhance many clinical use scenarios in diffuse and focal liver diseases.

## Data Availability

Not applicable.

## Abbreviations

AUC: Area under the curve
B-mode: Brightness-mode
CAP: Controlled attenuation parameter
CEUS: Contrast-enhanced ultrasound
CT: Computed tomography
DECT: Dual-energy CT
DWI: Diffusion-weighted imaging
HCC: Hepatocellular carcinoma
MRE: Magnetic resonance elastography
MRI: Magnetic resonance imaging
MRS: Magnetic resonance spectroscopy
NAFLD: Nonalcoholic fatty liver disease
NASH: Nonalcoholic steatohepatitis
PCD-CT: Photon-counting detector CT
pCT: Perfusion CT
PDFF: Proton density fat fraction
pSWE: Point shear wave elastography
TE: Transient elastography
US: Ultrasound
